# Effect of α-tocopherol in alleviating the lipopolysaccharide-induced acute lung injury via inhibiting nuclear factor kappa-B signaling pathways

**DOI:** 10.1080/21655979.2022.2031399

**Published:** 2022-02-03

**Authors:** Mu Hu, Jielai Yang, Yang Xu

**Affiliations:** Department of Orthopedics, Ruijin Hospital, Shanghai Jiao Tong, University School of Medicine, Shanghai, China

**Keywords:** α -tocopherol, acute lung injury, NF-κB signaling pathway

## Abstract

Acute respiratory distress syndrome (ARDS) leads to the acute lung injury (ALI), a form of diffused alveolars injury, accompanied by severe inflammation and oxidative damage of alveolar epithelial cells. α-Tocopherol (α-TOH), one of the eight isoforms of vitamin E, is a natural antioxidant-free radical. We aimed to understand the effect of α-TOH and mechanism involved in inducing the ALI. Lipopolysaccharide (LPS) is injected into the trachea of mice to generate ALI mouse models. α-TOH was used to administrate the mice intragastrically to detect the expression of inflammatory factors and antioxidant molecules by enzyme linked immunosorbent assay, hematoxylin–eosin staining and immunohistochemical staining. Mouse alveolar epithelial cell line (MLE-12 cells) was used to determine the effect of α-TOH on alveolar epithelial cells. Inflammatory factors such as, interleukin (IL)-1β, IL-6, and tumor necrosis factor (TNF)-α shows significant increase in the lung tissues of the mice induced by LPS and reduction in the expressions of superoxide dismutase (SOD)1/2 and glutathione peroxidase (GSH-Px). After treatment with α-TOH, the inflammation and oxidative stress levels shows substantial reduction in the lung tissues of the mice. Moreover, α-TOH also increases the proliferation ability of MLE-12 cells *in vitro* and reduces apoptosis level. In addition, α-TOH reduces p65 phosphorylation and nuclear translocation in alveolar epithelial cells *in vivo and in vitro*, thus, inhibiting the activity of the nuclear factor kappa-B (NF-κB) signaling pathway. α-TOH reduces the inflammation and oxidative stress of lung tissue by inhibiting the NF-κB signaling pathway, thereby alleviating the LPS-induced ALI.

## Graphical abstract

**Figure:**
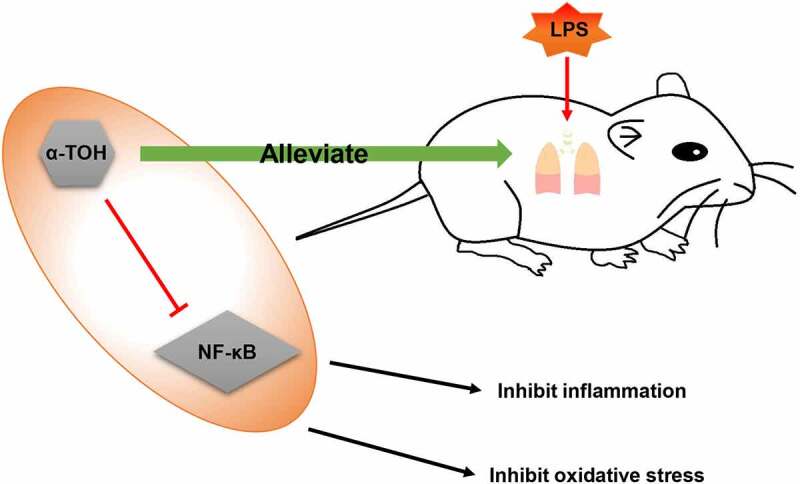

## Introduction

Acute respiratory distress syndrome (ARDS) lead to the acute lung injury (ALI), which is a syndrome characterized by acute progressive and refractory hypoxemia caused by various non-cardiogenic factors, such as severe infection, shock, trauma, or burn [1]. The main symptoms of ALI are the severe inflammatory reaction in the lungs that leads to the destruction of the alveolar endothelial barrier, the infiltration of neutrophils and other inflammatory cells accompanied by the release of inflammatory and cytotoxic media, and the activation of alveolar epithelial cells and effector T cells [2]. A large amount of inflammatory exudation causes the alveoli to be filled with protein-rich fluids thereby, affecting the respiratory function of the alveoli. The impaired synthesis and metabolism of surfactants further worsens the patient’s respiratory function [3]. In addition, the balance of oxidation and antioxidation in lung tissues is also damaged due to the destruction of a large number of inflammatory factors [4]. Therefore, for treatment of ALI and ARDS, it is important to take intervention measures to control the ‘waterfall’ effect of lung inflammation and oxidative stress, inhibiting the production of inflammatory factors and reducing the oxidative damage of alveolar epithelial cells.

α-tocopherol (α-TOH) is a type of vitamin E. Nair et al. [5] reported that vitamin E has good antioxidant properties while regulating endocrine system and promoting reproduction. Various available literature reported that α-TOH is a fat-soluble vitamin that penetrates in the cell membrane and reduces the generation of oxygen-free radicals [6]. In addition, α-TOH also plays an important role in inflammatory diseases. Wallert et al. [7] observed that in a mouse model of myocardial ischemia-reperfusion injuries, the α-TOH effectively reduces the inflammation level and oxidative stress level of myocardial tissue. α-TOH has also been found to reduce the levels of interleukin (IL) −1β and IL-6 in human gingival fibroblasts [8]. Therefore, α-TOH has a potential therapeutic effect on inflammation and oxidative damage. However, the role of α-TOH in ALI and ARDS is still unclear and detailed investigation is required. Therefore, in the present study, Lipopolysaccharide (LPS) is used to induce sepsis in normal mice and α-TOH treated mice to investigate the effect of α-TOH on LPS-induced ALI.

## Materials and methods

### Animals and grouping

C57BL6 male mice (8 weeks old, n = 60) were purchased and kept for further experimentation at the Experimental Animal Center of Ruijin Hospital, Shanghai. The mice were placed in SPF environment with proper sunshine and fed with healthy food and clean water. LPS (5 mg/kg, Sigma-Aldrich, St. Louis, MO, USA) was used to induce sepsis in mice [9,10]. For the experimentation, all mice were divided into different groups, such as control group, LPS group, LPS+low-dose α-TOH (L-α-TOH) group, and LPS+high-dose α-TOH (H-α-TOH) group. LPS stimulates the ALI in mice. After the mouse was anesthetized, LPS was injected into the neck trachea using micro syringe accompanied by immediate shook to distribute the LPS evenly in the body. The enlargement of wet rales in the chest of the mouse indicated that the LPS was successfully entered the alveoli. The mice in the groups, LPS+low-dose α-TOH (L-α-TOH) (100 mg/kg) group and LPS+high-dose α-TOH (H-α-TOH) (200 mg/kg) group, were administered with α-TOH (Selleck, Shanghai, China) intragastrically daily for 2 weeks before injecting LPS [11]. One day after LPS injection, the lung tissue of the mouse was collected for further experimentation.

### Wet/dry (W/D) weight ratio of lung tissue

After collecting the complete sample of lung tissue of the mouse, the wet weight of the lung tissue was measured. The lung tissue was then placed in an incubator at 60°C for 48 h to dry it up completely. Once the lung tissue was dried, the sample was weighed to calculate the W/D weight ratio. The W/D ratio represents the severity of lung tissue edema.

### Myeloperoxidase (MPO), malondialdehyde (MDA), superoxide dismutase (SOD) glutathione peroxidase (GSH-Px), and activity assay

The concentration of Myeloperoxidase (MPO), Malondialdehyde (MDA), Superoxide Dismutase (SOD), and Glutathione Peroxidase (GSH-Px) in mouse serum represents the level of oxidative stress in mice. After collecting the mouse serum, the manufacturer’s instructions given on the MPO kit (R&D Systems, Emeryville, CA, USA), MDA kit (R&D Systems, Emeryville, CA, USA), SOD kit (R&D Systems, Emeryville, CA, USA) and GSH-Px kit (R&D Systems, Emeryville, CA, USA) were followed to detect the oxidative stress level in the mouse.

### Preparation of bronchoalveolar lavage fluid (BALF)

The fresh mouse lung tissue was perfused using phosphate buffer solution (PBS) 3 times. After centrifuging the BALF, the supernatant and sediment was collected separately, to measure inflammation levels and cell counting, respectively. After resuspension, the collected cell pellet was spread on the glass slide and stain with Wright Giemsa to have clear cell and neutrophils counts under the microscope (LEICA, Koln, Germany).

### Enzyme linked immunosorbent assay (ELISA)

The supernatant is collected and impurities were removed by centrifugation. All samples (including samples to be tested and standards) were added to a special 96-well plate according to the ELISA kit (R&D Systems, Emeryville, CA, USA) protocol. We draw a standard curve based on the concentration of the standard and the measured absorbance, and calculated the concentration based on the absorbance of the sample to be tested.

### Hematoxylin-eosin (HE) staining

Mouse lung tissue was fixed in 4% paraformaldehyde solution for 24 h and later made into the paraffin block. Then, the paraffin block was cut into 5 μm thick slices and attached to glass slides. After baking the slices in incubator at 37°C for 3 days, hematoxylin and eosin staining (Beyotime, Shanghai, China) used to stain the cell nucleus and cytoplasm of lung tissue, respectively. Finally, the stained slides were recorded using optical microscope.

### Immunohistochemical (IHC) staining

After dewaxing and hydration, the slices were dipped in citrate buffer and heated to 95°C for 20 min. After that, 3% H_2_O_2_ was used to inactivate peroxidase. Then, nonspecific antigens were blocked with 10% goat serum and subjected to the incubation of lung tissue at 4°C overnight with primary antibody dilution (IL-1β, ab2105, Abcam, Cambridge, MA, USA; TNF-α, ab1793, Abcam, Cambridge, MA, USA; SOD1, ab13498, Abcam, Cambridge, MA, USA; SOD2, ab68155, Abcam, Cambridge, MA, USA; p-p65, ab86299, Abcam, Cambridge, MA, USA). After washing the slices with PBS, the lung tissue was incubated with secondary antibody dilution (Abcam, Cambridge, MA, USA) for another 1 h. Finally, DAB was used for color development and the staining results were observed using optical microscope.

### Cell culture and treatment

The mouse alveolar epithelial cell line (MLE-12 cell) was used in the present study. Dulbecco’s modified eagle medium (DMEM) (Gibco, Rockville, MD, USA) containing 10% fetal bovine serum (Gibco, Rockville, MD, USA) and 1% penicillin plus streptomycin (Gibco, Rockville, MD, USA) used to culture MLE-12 cells in an incubator at 37°C and 5% CO_2_. LPS was used to induce MLE-12 cell injury.

### Cell counting kit-8 (CCK8) assay

MLE-12 cells were seeded in 96-well plates. After treating cells with LPS and α-TOH for 24 h, 10 μL of CCK8 reagent (Dojindo Molecular Technologies, Kumamoto, Japan) was added to the each well of 96-well plate and incubated at 37°C for 1 h. Then, a microplate reader was used to detect the absorbance of each well at 450 nm. The blank group carried only the medium without the presence of any cells whereas, and the cells without treatment were present in the control group.

Cell viability = (OD_sample_-OD_blank_)/(OD_control_-OD_blank_)

### 5-ethynyl-2’-deoxyuridine (EdU) cell proliferation assay

The cell proliferation level were detected by EdU cell proliferation assay. EdU staining stains only the nucleus of cells during proliferating stage without staining rest components of the cell. The manufacturer instructions given on the EdU kit (Sigma-Aldrich, St. Louis, MO, USA) was used to detect the effect of α-TOH on the proliferation ability of MLE-12 cells.

### Terminal deoxynucleotidyl transferase (TdT) -mediated dUTP-biotin nick end labeling (TUNEL) assay

The TUNEL assay was used to detect the apoptosis level in MLE-12 cells. In apoptosis, a large number of 3’-OH ends are produced by chromosomal DNA double-strand breaks or single-strand breaks. The 3’-OH terminal is used to tag deoxyribonucleotide and luciferin, peroxidase, alkaline phosphatase or biotin derivatives to the 3’-terminal of DNA under the action of TdT. TUNEL kit (Sigma-Aldrich, St. Louis, MO, USA) detected the apoptosis level of MLE-12 cells according to the manufacturer’s instructions.

### Western blot

Mouse lung tissue and MLE-12 cells were lysed by RIPA lysate. Mouse lung tissue needs to be additionally put into a homogenizer to fully lyse. The protein lysate was then centrifuged (12,000 g, 15 min, 4°C) and the supernatant was collected. After being mixed into the loading buffer, the protein lysate was heated to 100°C for 5 min. Then, we made a 10% sodium dodecyl sulfate polyacrylamide gel and added 10 μL of protein lysate. After electrophoresis and membrane transfer, the protein is transferred to the polyvinylidene fluoride membrane. We used 5% bovine serum albumin-PBS-Tween to block nonspecific antigens for 1 h at room temperature. The membrane was then incubated with primary antibody dilutions (β-actin, ab8226, Abcam, Cambridge, MA, USA; IL-1β, ab254360, Abcam, Cambridge, MA, USA; tumor necrosis factor (TNF), ab1793, Abcam, Cambridge, MA, USA; SOD1, ab51254, Abcam, Cambridge, MA, USA; SOD2, ab68155, Abcam, Cambridge, MA, USA; caspase 3, ab184787, Abcam, Cambridge, MA, USA; p65, ab16502, Abcam, Cambridge, MA, USA; p-p65, ab86299, Abcam, Cambridge, MA, USA; IκBα, ab183503, Abcam, Cambridge, MA, USA;) at 4°C overnight. After washing the membrane, we incubated the membrane at room temperature for 1 h with the secondary antibody dilution (Abcam, Cambridge, MA, USA). Finally, we used chemiluminescent liquid for color development.

### Immunocytofluorescence (IF) staining

After washing the cells with PBS, the cells were treated sequentially with 4% paraformaldehyde and 0.2% Triton X-100. Then, blocks containing cells were made with 10% goat serum and incubated at 4°C for overnight with primary antibody dilution (p65, ab16502, Abcam, Cambridge, MA, USA). After washing the residual primary antibody with PBS, cells were incubated using fluorescent secondary antibody dilution (Abcam, Cambridge, MA, USA) in the dark for another 1 h. The cell nucleus was stained using DAPI. Finally, the staining results were recorded under fluorescence microscope.

### Statistical analyses

Statistical Product and Service Solutions (SPSS) 21.0 statistical software was used to analyze the data of the present investigation. All data measurements were represented as mean ± standard deviation. T-test and one-way analysis of variance were used to compare between different groups. *p* < 0.05 was consider statistically significant. All experiments were repeated three times to remove the errors, if any.

## Results

### α-TOH reduces LPS-induced inflammation in lung tissue of mice

**Figure 1.**
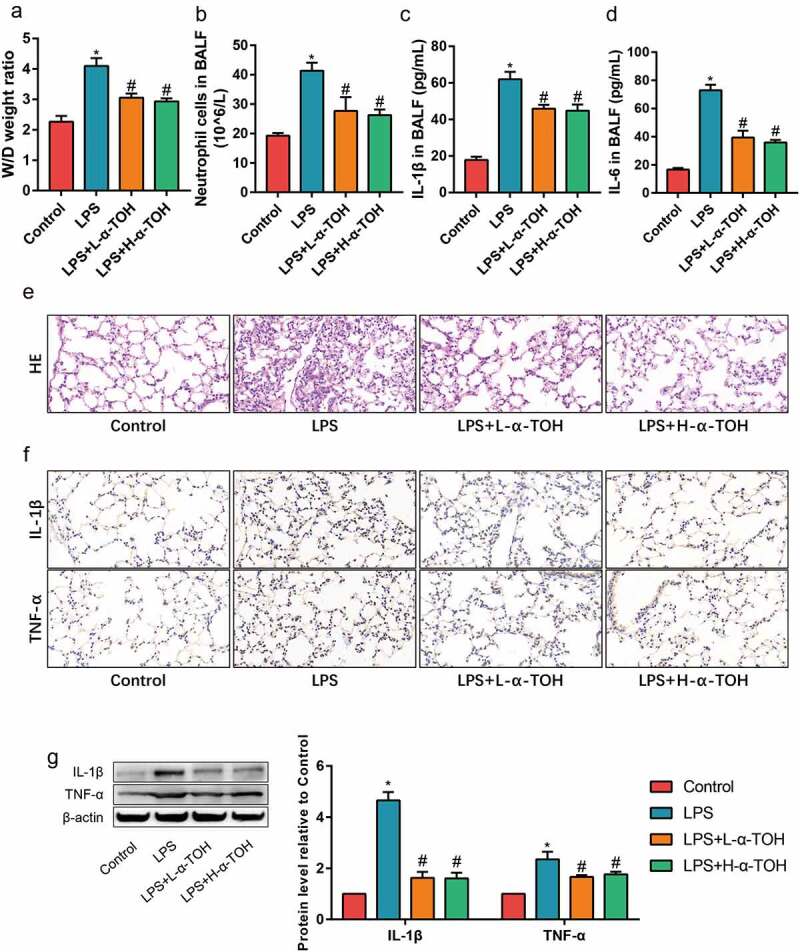
α-TOH reduces LPS-induced inflammation in lung tissue of mice treated with low-dose α-TOH and high-dose α-TOH.

To determine the activity of α-TOH on LPS-induced ALI, the inflammation level of mouse lung tissue divided into various groups were examined and well-studied in detail. The degree of edema in the lung tissue of mice was obtained after measuring the W/D weight ratio (Figure 1(a)). It was observed that the W/D weight ratio of the lung tissue of mice in the LPS group was higher than that of the control group, whereas the edema of the lung tissue significantly improved after treatment with α-TOH. The total number of neutrophils in BALF for different mice by cell counts were also compared to look upon the test results. It’s been observed that the number of neutrophils in BALF of the LPS group showed higher compared to the control group, and α-TOH also reduced the infiltration of inflammatory cells in lung tissue (Figure 1(b)). The inflammatory factors (IL-1β and IL-6) in BALF by ELISA was also examined. α-TOH significantly reduced the concentration of IL-1β (Figure 1(c)) and IL-6 (Figure 1(d)) in BALF. HE staining examined the morphological changes in mouse lung tissue and it showed that the lung interstitial edema of the mice and infiltration of inflammatory cells were found in the LPS group and α-TOH treatment improved the structure of the lung tissue (Figure 1(e)). The results of IHC staining also showed that α-TOH treatment reduced the expression of IL-1β and TNF-α in lung tissue (Figure 1(f)). In addition, the results of Western blot were also consistent with IHC staining (Figure 1(g)). These results revealed that α-TOH reduced the inflammation level in LPS-induced lung tissue, whereas no significant difference is achieved with the high and low α-TOH concentrations.

(a) Wet/dry weight ration of mice; (b) Number of neutrophil cells in BALF of mice; (c, d) ELISA results of IL-1β and IL-6 in BALF of mice; (e) HE staining results of mice lung in four groups (400×); (f) IHC staining results of IL-1β and TNF-α in mice lung (400×); (g) Western blot results of IL-1β and TNF-α. There is no statistical difference in the therapeutic effect of low-dose α-TOH and high-dose α-TOH. (‘*’ means *p* < 0.05 vs. control group and ‘#’ means *p* < 0.05 vs. LPS group)

### α-TOH reduces LPS-induced oxidative stress in lung tissue of mice

**Figure 2.**
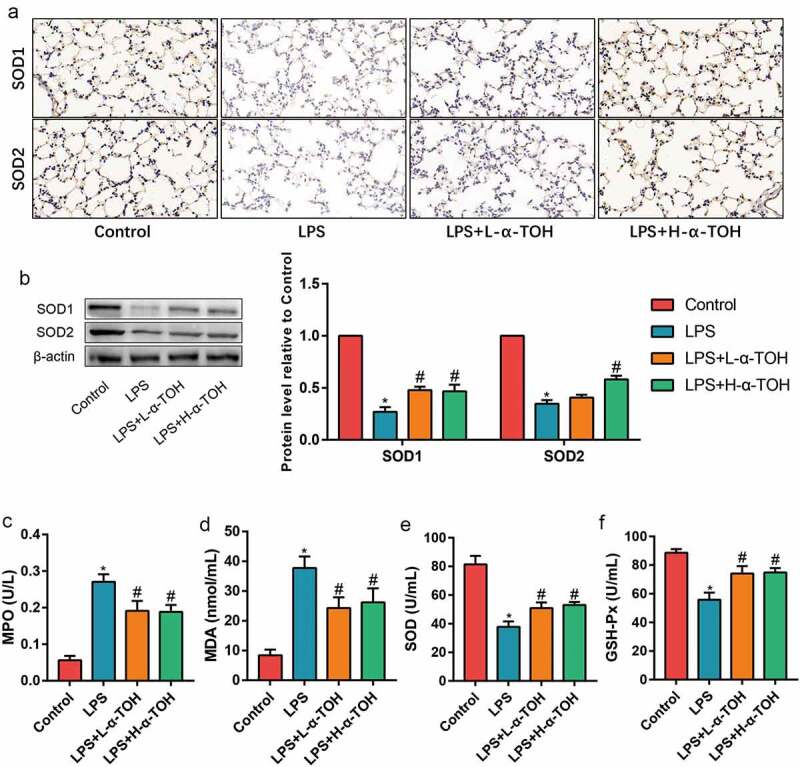
α-TOH reduces LPS-induced oxidative stress in lung tissue of mice. (a) IHC staining results of SOD1 and SOD2 in mice lung (400×); (b) Western blot results of SOD1 and SOD2; (c–f) The concentration of MPO, MDA, SOD, and GSH-Px in mice serum. (‘*’ means *p* < 0.05 vs. control group and ‘#’ means *p* < 0.05 vs LPS group)

The increment in the oxidative stress level is also one of the pathogenic factors of ALI. SOD1/2 expression were identified using IHC staining (Figure 2(a)) and Western blot (Figure 2(b)). The α-TOH dosage used for these experiments was the same as above. The expression of SOD1/2 in the lung tissues of mice in the LPS group considerably decreased, indicating that LPS caused decrease in the antioxidant capacity of the lung tissue in the mice. After treating mice with α-TOH, the expression of SOD1/2 in the lung tissue of the mice significantly increased. In addition, the levels of MPO, MDA, SOD, and GSH-Px were also identified in the serum of mice and observed that the levels of SOD and GSH-Px in the serum of mice for LPS group decreases, while the level of MPO and MDA increased, implying the damage to the lung tissue reduced the antioxidant capacity (Figure 2(c–f)). After treating mice with α-TOH, the antioxidant capacity of the lung tissue of mice improved significantly.

### α-TOH alleviates LPS-induced MLE-12 cell injury

**Figure 3.**
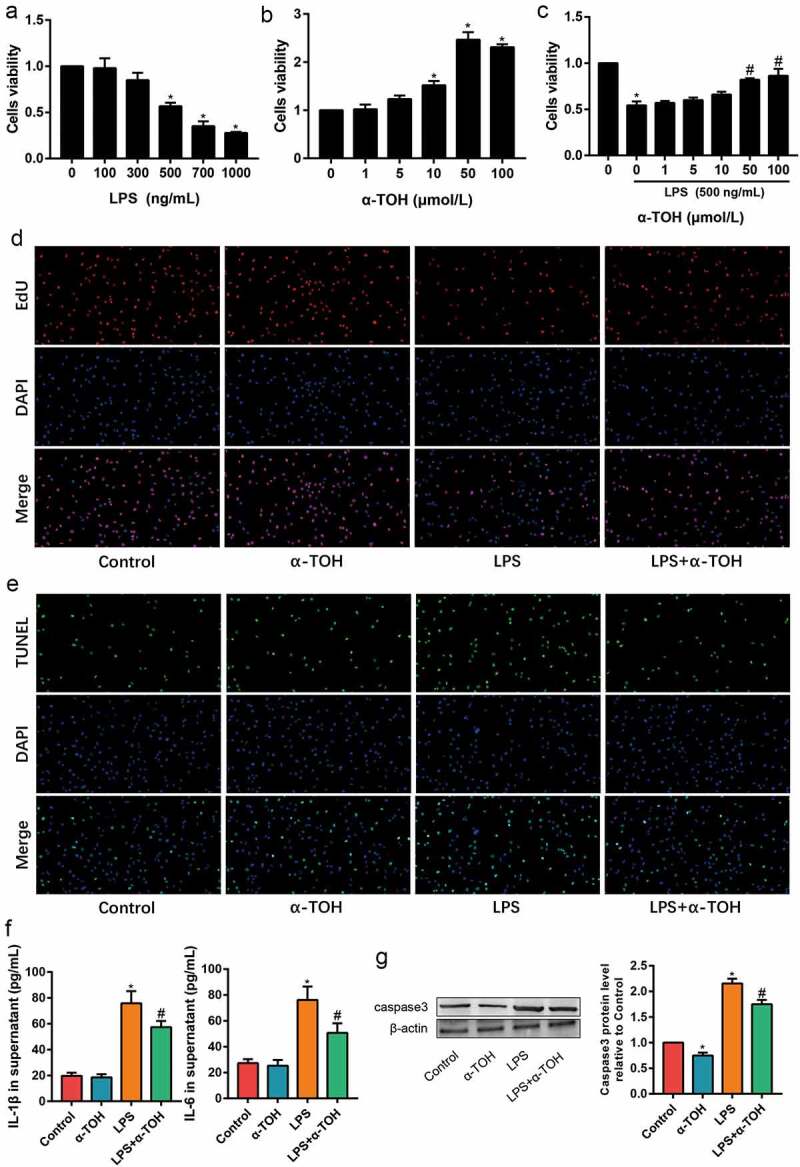
α-TOH alleviates LPS-induced MLE-12 cell injury. (a, b) Effect of LPS and α-TOH on proliferation ability of MLE-12 cells; (c) Effect of α-TOH on proliferation ability of MLE-12 cells on the basis of LPS; (d) Results of EdU cell proliferation assay in MLE-12 cells (200×); (e) Results of TUNEL assay in MLE-12 cells (200×); (f) ELISA results of IL-1β and IL-6 in the cell supernatant; (g) Western blot results of caspase 3. (‘*’ means *p* < 0.05 vs control group and ‘#’ means *p* < 0.05 vs LPS group)

In order to further clarify the effect of α-TOH on alveolar epithelial cells, we used α-TOH to stimulate MLE-12 cells and examined the activity of MLE-12 cells *in vitro*. The variable concentrations, such as 100, 300, 500, 700, and 1000 ng/mL of LPS, were used for the toxicity examination of MLE-12 cells through the CCK8 assay. In the results for CCK8 assay, its observed that the 500 ng/mL of LPS drastically caused damage to MLE-12 cells (Figure 3(a)). In addition, the optimal concentration of α-TOH on MLE-12 cells was determined through CCK8 assay and found that 50 μmol/L of α-TOH significantly increased the viability of MLE-12 cells (Figure 3(b,c)). The result of EdU staining also proved that α-TOH increased the proliferation ability of MLE-12 cells (Figure 3(d)). TUNEL assay detected the level of MLE-12 cells apoptosis. The apoptosis level of LPS-induced MLE-12 cells was higher than that of control group, while α-TOH decreases the percentage of apoptotic cells (Figure 3(e)). The inflammation factors in the cell supernatant were examined using ELISA and the results revealed that IL-1β and TNF-α increased in the LPS group and α-TOH can reduce their expression (Figure 3(f)). In addition, the cell apoptosis maker, caspase 3, was examined using Western blot and the result showed that α-TOH can reduce the expression of caspase 3 (Figure 3(g)).

### α-TOH inhibits NF-κB signaling pathway in alveolar epithelial cells in vivo and in vitro

**Figure 4.**
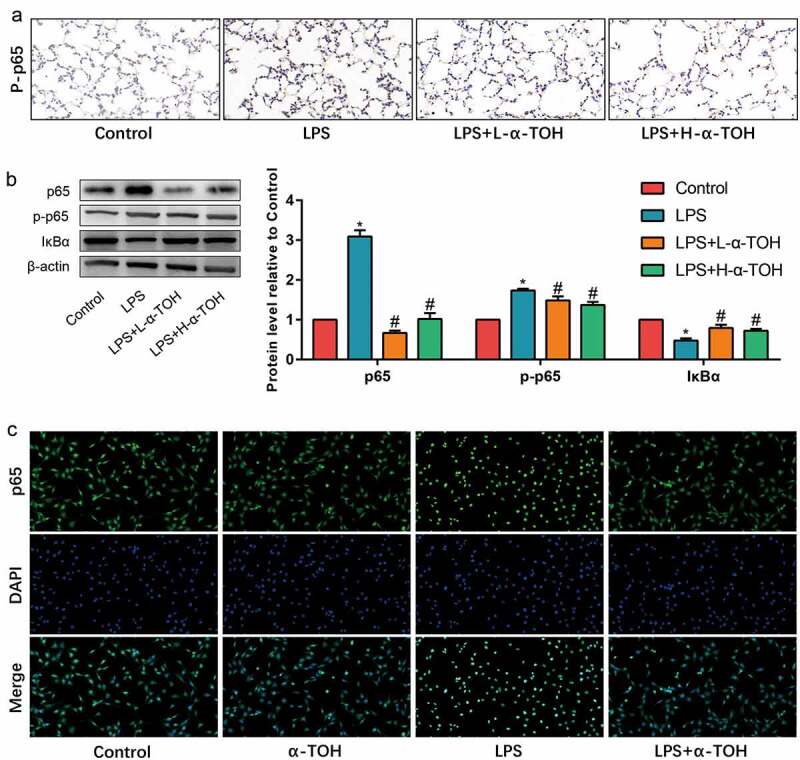
α-TOH inhibits NF-κB signaling pathway in alveolar epithelial cells in vivo and in vitro. (a) IHC staining results of p-p65 in mice lung (400×); (b) Western blot results of p65, p-p65, and IκBα; (c) IF staining results of p65 in MLE-12 cells.

The NF-κB signaling pathway is one of the important inflammation-related pathways, which is used to determine the changes undergone in alveolar epithelial cells on utilizing NF-κB signaling pathway. The expression level of p65, p-p65, and IκBα in mouse lung tissue was detected by IHC staining and Western blot. The expression of p65 and p-p65 in the lung tissue of mice in the LPS group shows significant increase, while α-TOH treated tissues shows decrease in the expression of p65 and the phosphorylation of p65 (Figure 4(a, b)). However, the result of IκBα was opposite (Figure 4(b)). In addition, the effect of α-TOH on p65 nuclear transfer *in vitro* was also detected and the results showed that after stimulating MLE-12 cells with LPS, p65 transferred to the nucleus of the cells. However, after treatment with α-TOH, the nuclear transfer of p65 was subsequently inhibit (Figure 4(c)). These results indicated that the NF-κB signaling pathway was activated in LPS-treated mouse lung tissue and MLE-12 cells, while α-TOH reduced the activity of the NF-κB signaling pathway.

## Discussion

ALI/ARDS refers to acute diffuse lung injury and acute respiratory failure cause due to various intrapulmonary and extrapulmonary pathogenic factors [12]. When lung tissue stimulates contrarily inside and outside the lung, a large number of neutrophils accumulate in the alveolar capillary endothelium under the action of chemokines (such as TNF-α, IL-8, LPS, complement 5A, leukotriene B4, thrombotin A2) and platelet activating factors, that releases the oxygen free radical and inflammatory mediators, leads to inflammatory and oxidative damage [13]. α-TOH, as an antioxidant, plays an important role in LPS-induced mouse ALI. The ALI mice samples treated with α-TOH, reduced the expression of IL-1β, IL-6, and TNF-α in the lung tissue and increased the expression of antioxidant enzymes SOD, MPO, and GSH-Px. In addition, α-TOH also increased the viability of mice alveolar epithelial cells and MLE-12 cells *in vitro*. The NF-κB signaling pathway was consider as an important alleyway for the existence and development of ALI [14]. The inhibitory effect of α-TOH on the NF-κB signaling pathway also revealed its protective effect against LPS-induced alveolar epithelial cell injuries.

The reasons for the development of ALI and ARDS include chemical factors (such as inhalation of poisonous gas, smoke, and stomach contents), physical factors (such as radiation damage), biological factors (such as severe acute respiratory syndrome) and systemic pathological processes (such as shock, extensive burns, and sepsis) [15]. Among them, sepsis is the most common cause of ALI and ARDS [16]. LPS, main component of the cell wall of gram-negative bacilli, is an important group-specific antigen of endotoxin [17]. LPS stimulates the inflammatory cells via activating multiple intracellular signal transduction pathways, such as NF-κB, MAPK, JAK/STAT, etc., resulting in the origination of large number of inflammatory mediators [18]. LPS acts directly on lung tissue through airway instillation and activates the inflammatory cells in the lung tissue to release pro-inflammatory cytokines, causing lung tissue injury [19]. Therefore, LPS is the most commonly used substance for designing endotoxin-induced ALI models. From histological staining, its been observed that the lung tissue of LPS-induced ALI model mice shows serious histopathological changes, including the destruction and collapse of some alveolar structures, thickening of the alveolar interstitium, and the infiltration of inflammatory cells, indicating LPS successfully induced ALI in mice sample. Moreover, α-TOH not only improved the structure of lung tissue, but also reduced the infiltration of inflammatory cells and the expression of inflammatory factors in the lung. In addition, α-TOH also improved the antioxidant capacity of lung tissue, thus, reducing the damage of alveolar epithelial cells caused by oxygen-free radicals.

Vitamin E, including α, β, γ, and δ-TOH [20], has very good antioxidant properties and is a kind of natural antioxidant-free radical substance [21]. Among all of them, α-TOH is the currently acknowledge strong antioxidant-free radical substance. It can compete with free radicals for unpaired electrons to form α-tocopherone, which is nontoxic to the human body. α-tocopherone effectively eliminates the toxicity of oxygen-free radicals of the cells, thereby playing a protective role [22]. The present investigation results represent that α-TOH plays pivotal antioxidant role for multiple disease models and cells. Herbet et al. [23] found that α-TOH alleviates the chronic variable stress-induced inflammation by improving redox balance. Shukla et al. [24] pretreated the rat model of myocardial infarction with α-TOH and observe that α-TOH attenuate the expression of inflammatory factors and cardiomyocyte apoptosis in rat myocardial tissues. The protective effect of α-TOH on lung tissue has also been studied for a long time. Ostojic et al. [25] used α-TOH and ascorbic acid to treat patients with early diffuse systemic sclerosis and observe that α-TOH improves the patient’s lung function. Hanson et al. [26] also observe a positive correlation between vitamin E intake and lung function. Therefore, α-TOH has a potential protective effect on the lungs. The results obtained in the present study also showed that α-TOH promotes the vitality of alveolar epithelial cells and reduces the level of apoptosis. The NF-κB signaling pathway is involved in the gene regulation of many inflammatory factors and plays an important role in ALI. α-TOH inhibited p65 phosphorylation and nuclear translocation, thereby, inhibiting the NF-κB signaling pathway. This might be one of the potential mechanisms of α-TOH protect the lung from any kind of injuries.

To sum up, this is the first study to investigate the therapeutic effect of α-TOH on ALI. It has been believed that the obtained results in the present study provides new research directions and targets for the clinical treatment of ALI.

## Conclusion

α-TOH played a potential lung protective role in LPS-induced ALI. α-TOH reduced the expression of inflammatory factors (IL-1β, IL-6, and TNF-α) and increased the expression of antioxidant molecules (SOD1/2 and GSH-Px) in lung tissue. In addition, α-TOH promoted the viability of mouse alveolar epithelial cells (MLE-12 cells) *in vitro* and reduced the level of apoptosis. The inhibitory effect of α-TOH on the NF-κB signaling pathway may be one of the mechanisms for exerting lung protection.

## Highlights

- α-TOH Reduces LPS-Induced Inflammation in Lung Tissue of Mice;
- α-TOH Reduces LPS-Induced Oxidative Stress in Lung Tissue of Mice;
- α-TOH Alleviates LPS-Induced MLE-12 Cell Injury;
- α-TOH Inhibits NF-κB Signaling Pathway in Alveolar Epithelial Cells *in vivo* and *in vitro*.
